# Polyurethane Foams Functionalized with Hydroxylated PAMAM G3 Dendrimers for Local Drug Delivery, Including Anticancer Therapy: In Vitro and In Vivo Studies

**DOI:** 10.3390/ma19214486

**Published:** 2026-10-24

**Authors:** Magdalena Zaręba, Magdalena Twardowska, Elżbieta Chmiel-Szukiewicz, Jakub Czechowicz, Dawid Łysik, Marcin Kuśmierz, Magdalena Rzepna, Jarosław Bieniaś, Justyna Bucior, Łukasz Uram

**Affiliations:** 1 Faculty of Chemistry, Rzeszow University of Technology, 6 Powstańców Warszawy Ave., 35-029 Rzeszów, Poland; magzar@prz.edu.pl (M.Z.); szukiela@prz.edu.pl (E.C.-S.); 176554@stud.prz.edu.pl (J.C.); justynanoga258@gmail.com (J.B.); luram@prz.edu.pl (Ł.U.); 2 Institute of Biomedical Engineering, Bialystok University of Technology, Wiejska 45C, 15-351 Białystok, Poland; d.lysik@pb.edu.pl; 3 Faculty of Chemistry, Maria Curie-Skłodowska University, 3 M. Curie-Skłodowska Sq., 20-031 Lublin, Poland; marcin.kusmierz@umcs.pl; 4 Centre for Radiation Research and Technology, Institute of Nuclear Chemistry and Technology, Dorodna 16, 03-195 Warsaw, Poland; m.rzepna@ichtj.waw.pl; 5 Department of Materials Engineering, Faculty of Mechanical Engineering, Lublin University of Technology, Nadbystrzycka 36, 20-618 Lublin, Poland; j.bienias@pollub.pl

**Keywords:** polyurethane foam, hydroxylated PAMAM dendrimer, doxorubicin, drug retention, dendrimer-modified polyurethane, SCC-15 and HaCaT, in vitro and in vivo studies, *Caenorhabditis elegans*

## Abstract

There is growing interest in local drug delivery systems that release their payload in a controlled manner directly at the implantation site. Such systems are particularly valuable in solid tumors, chronic wounds and infections, where systemic administration is constrained by poor tissue penetration and dose-limiting side effects. In this study, hydroxylated poly(amidoamine) (PAMAM) G3 dendrimers were incorporated into polyurethane foams (PU) as multifunctional polyol components to obtain hybrid porous matrices. Surface polyhydroxylation of amine-terminated PAMAM G3 via glycidol ring-opening introduced hydroxyl functionalities that enabled integration of the modified dendrimer into the PU network. Morphological analysis by scanning electron microscopy (SEM) and micro-computed tomography (µCT) confirmed a well-defined cellular architecture without macroscopic defects upon dendrimer incorporation. The modified material exhibited reduced apparent density and increased water absorption compared with the reference PU foam. Rheological creep–recovery analysis demonstrated enhanced delayed elasticity and reduced permanent deformation in the dendrimer-modified matrix, reflecting improved viscoelastic recovery. Mass balance analysis showed higher encapsulation efficiency for the dendrimer-modified foam (34.9%) than for the reference material (29.7%). Despite this higher loading, the cumulative release of doxorubicin (DOX) over 100 h in phosphate-buffered saline (PBS) (pH 7.4, 37 °C) was lower for the modified system (7.0%) than for the reference matrix (15.8%), and the initial burst release after 30 min was six-fold lower, indicating enhanced drug retention and the potential for prolonged release. Biological studies showed that the matrices were non-toxic and highly biocompatible towards human SCC-15 and HaCaT cells in vitro and in the *Caenorhabditis elegans* model in vivo, whereas DOX released from the matrices effectively killed cancer cells at extract concentrations as low as 3%. These results show that hydroxylated PAMAM G3 acts as a structural modifier of PU foams capable of modulating drug retention and release, and therefore use of dendrimeric matrix as a drug carrier in local therapy is worth considering.

## 1. Introduction

Localized drug delivery aims to maintain therapeutic concentrations of an active compound within a defined tissue volume while minimizing systemic exposure. This approach is particularly relevant in the management of solid tumors, post-resection cavities, chronic wounds and implant-associated infections, where systemic administration is constrained by limited tissue penetration, rapid clearance and dose-limiting toxicity [1]. Doxorubicin (DOX) exemplifies this problem: despite its broad anticancer efficacy, its clinical use is restricted by cumulative, dose-dependent cardiotoxicity, attributed to ferroptosis as a central mechanism. DOX disrupts myocardial iron homeostasis and impairs key antioxidant defense systems. At the same time, it reprograms lipid metabolism towards the accumulation of peroxidation-prone substrates, and the resulting excessive lipid peroxidation culminates in ferroptotic cell death [2]. Confining DOX release to the target site is therefore a long-standing therapeutic objective, and one that has driven the development of a broad range of carrier platforms [3].

The currently available local delivery platforms nevertheless remain imperfect, and each class fails on a different axis. Injectable colloidal carriers, such as liposomes, polymeric micelles and dendrimer–drug conjugates, achieve high loading and tunable release in solution, but they are not retained at the administration site and provide no mechanical support to the surrounding tissue [1,4]. Hydrogels, including thermoresponsive and in situ gelling systems, offer excellent biocompatibility and straightforward loading of hydrophilic drugs. However, their high water content and large mesh size usually translate into rapid, diffusion-dominated release, and their mechanical strength is insufficient for load-bearing or cavity-filling applications [5,6]. Inorganic carriers such as calcium phosphate cements provide mechanical integrity and are compatible with low-temperature drug incorporation, but their release profiles and resorption rates are difficult to match to therapeutic timescales [7]. A single material class that simultaneously offers site retention, a mechanically stable and shapeable porous architecture, non-acidic degradation behavior and chemically tunable drug affinity is therefore still lacking.

Polyurethanes (PUs) address several of these constraints. Their properties can be adjusted across a wide range, from elastomeric to rigid, by selecting the polyol, the diisocyanate and the crosslinking conditions [8,9]. Polyurethanes are therefore well established in cardiovascular devices, wound dressings, coatings and tissue engineering scaffolds [10,11]. Their segmented block-copolymeric character, and the resulting control over both physical properties and degradation behavior, underlies this versatility [12]. The same structure–property–function relationships have been exploited specifically to regulate drug release [13]. Processing into porous foams adds an interconnected cellular architecture that permits fluid penetration, mass transport and tissue ingrowth, and allows the material to be shaped to fill an irregular cavity [14,15,16]. In such systems the polymer network functions simultaneously as a structural framework and as a diffusion-controlling phase [17]. Critically, PU transport behavior is governed by parameters accessible to synthetic control: pore size and interconnectivity, apparent density, surface polarity, crosslink density and water uptake [18,19]. What conventional polyether-polyol-based PU foams lack, however, is specific chemical affinity for the drug. Release from them is essentially a partition–diffusion process, and retention is short. Introducing affinity sites into the network, rather than merely adsorbing the drug onto it, is thus the key missing element.

Poly(amidoamine) (PAMAM) dendrimers provide exactly this functionality. Their monodisperse, generation-defined architecture combines internal cavities capable of hosting guest molecules with a high density of peripheral functional groups [20,21,22]. DOX in particular has been widely studied in PAMAM-based carriers, either as non-covalent complexes or as conjugates with native and modified dendrimers [23,24,25]. Their use as free carriers is nevertheless limited by the same retention problem that affects other colloidal systems. In addition, amine-terminated dendrimers of higher generations display concentration-dependent cytotoxicity arising from their cationic surface charge [26,27,28]. Consistently, hydroxyalkylation of the dendrimer periphery with glycidol has been shown to yield highly biocompatible constructs that do not impair proliferation or mitochondrial activity in vitro, nor viability in *Caenorhabditis elegans* and rat models in vivo [29]. Covalent immobilization within a solid matrix offers a route around both limitations simultaneously: the dendrimer is retained at the implantation site together with the scaffold, and its cationic character is substantially reduced by the hydroxylation step that precedes immobilization.

We previously demonstrated that PAMAM G3 can be introduced into PU foams based on polyether polyol and castor oil, and that it modulates DOX loading and release [30]. However, foams with high dendrimer content (≥30 wt%) lose structural integrity, showing irregular cells, pore coalescence and cell-wall thinning, while releasing low-molecular-weight species containing PU and PAMAM components as well as PAMAM–DOX complexes into the surrounding medium [31]. The present work addresses this limitation by converting the dendrimer periphery prior to foaming. Reaction of amine-terminated PAMAM G3 with an excess of glycidol proceeds by epoxide ring-opening at the terminal amines. Each of the 32 primary amine groups adds two glycidol molecules, giving a total of 64 1,2-dihydroxypropyl substituents bearing 128 hydroxyl groups, and converting the periphery to tertiary amines [32]. The resulting hydroxylated dendrimer (PAMAM G3^gl^) is a water-soluble multifunctional polyol whose hydroxyl groups can react with isocyanate during foam formation. The dendrimer is thereby incorporated as a branched node of the polyurethane network rather than as a dispersed additive, while its interior amide and tertiary amine layers, responsible for DOX binding, remain available.

Generation 3 was selected as a deliberate compromise. It provides sufficient internal volume and terminal group density to interact with DOX while retaining the miscibility with polyether polyols required for homogeneous mixing and uniform cell nucleation. Dendrimers of generations 3 to 5 are, moreover, the range most commonly exploited as drug carriers [32]. Higher generations introduce greater steric congestion and a larger mismatch with conventional polyols, which compromises processability and cellular uniformity. Lower generations, in turn, offer too few functional groups to act effectively as network nodes.

We hypothesized that incorporation of hydroxylated PAMAM G3 into a PU foam would increase the polarity, hydrophilicity and crosslink density of the network. We further expected these changes to jointly enhance DOX encapsulation while slowing its release relative to an unmodified reference foam. To test this, the hybrid PU–PAMAM G3^gl^ foams were characterized with respect to cellular morphology using scanning electron microscopy (SEM) and micro-computed tomography (µCT), apparent density and water absorption, viscoelastic creep–recovery behavior, surface composition using X-ray photoelectron spectroscopy (XPS), and DOX loading and release kinetics under physiological conditions. Biological performance was evaluated on immortalized human keratinocytes (HaCaT) and squamous cell carcinoma cells (SCC-15) in vitro, and in the *Caenorhabditis elegans* model in vivo. Moreover, in vitro skin irritation test using a reconstructed human epidermis model was performed. The study thus tests a material-centered strategy in which the dendrimer operates not as a free nanocarrier but as an integrated structural modifier that reshapes the transport properties of the host network.

## 2. Materials and Methods

### 2.1. Materials

Rokopol^®^ G1000 and Rokopol^®^ G441, two polyoxyalkylene triols used in this study, were obtained from PCC Rokita (Brzeg Dolny, Poland). 4,4′-Diphenylmethane diisocyanate (mixture of di- and triisocyanates) for synthesis (pMDI, Merck Life Science, Darmstadt, Germany); Triethylamine (TEA, 99.5%, Chempur, Piekary Śląskie, Poland); Silicone PU-8580 (Silibase, Jiande, China); Glycerol (Gly, 99.5%, Sigma-Aldrich, Saint Louis, MO, USA); Ethanol (Supelco, Bellefonte, PA, USA); Glycidol (Gl, 96%, mixture of enantiomers, Thermo Fisher Scientific, Waltham, MA, USA); Doxorubicin hydrochloride (DOX, Pol-Aura, Zawroty, Poland); Phosphate-Buffered Saline (PBS) was purchased from Sigma-Aldrich, Saint Louis, MO, USA). The polyhydroxylated PAMAM G3 dendrimer (G3^gl^) was obtained by reacting a third-generation PAMAM dendrimer with glycidol, following the procedure described in our earlier work [32]. PAMAM G3 dendrimer was obtained according to Tomalia et al. and purified according to Esfand and Tomalia [20].

### 2.2. Synthesis of Polyurethane Foams Modified with Hydroxylated PAMAM G3

The polyol mixture was prepared in a 200 cm^3^ polypropylene cup by combining PAMAM G3^gl^, glycerol, and glycerin-based polyoxyalkylene triol G1000 in the proportions specified in Table 1. The G1000 polyol was characterized by a hydroxyl number of 155–165 mg KOH/g and a dynamic viscosity of 200–300 mPa·s at 25 °C. Homogenization was carried out at 50 °C until a uniform polyol–dendrimer blend was obtained. Before foam preparation, the resulting mixture was maintained at room temperature for five days. Following conditioning, water and silicone surfactant were incorporated into the mixture in the amounts specified in Table 1, with stirring at approximately 45 °C. After cooling to room temperature, the calculated quantity of polymeric methylene diphenyl diisocyanate (pMDI), determined using Equation (1), was introduced together with triethylamine (TEA) as a catalyst. Vigorous mixing was continued until foaming began. After free expansion, the resulting foams were left at room temperature for a minimum of two weeks before characterization. The reference foam (G) was prepared following the same general procedure, except that its polyol components were combined directly at room temperature, omitting the initial heating and five-day conditioning stages.

(1) $$m_{pMDI}=\left[\frac{100\;\cdot \;42}{p_{NCO}}\cdot \left(\sum_{i=1}^{n}\frac{m_{i\;}f_{i}}{M_{i}}+\frac{2m_{H_{2}O}}{18}\right)\right]\cdot k$$

where *m*_pMDI_ is the mass of polymeric methylene diphenyl diisocyanate (pMDI) [g], *p*_NCO_—content of NCO groups in pMDI [wt%], 42—molar mass of the NCO group, *m_i_*—mass of the individual polyol component (G3^gl^, G1000 or glycerol) used in the foaming process [g], *f_i_*—functionality of the corresponding polyol component, *M_i_*—molar mass of the polyol component, $m_{H_{2}O}$_—_mass of water, 18—molar mass of water, *k*—isocyanate index was 0.8 for both PU foams (G and G3^gl^G).

### 2.3. Analytical Methods

#### 2.3.1. Apparent Density and Water Absorption

The apparent density (*ρ*_bulk_) the investigated foams was calculated from the mass (m) and geometric volume (V) of cubic specimens, following UNE EN ISO 845:2010 [33].

Water uptake was evaluated according to ISO 2896:2001 [34]. Foam specimens were immersed in distilled water for three exposure periods: 5 min, 3 h and 24 h. The volumetric water absorption (%) was then determined using Equation (2).

(2) $$\%W_{A}=\frac{m_{2}-m_{1}}{V_{0}\times d_{W}}\times 100\%$$

where *m*_1_ and *m*_2_ represent the specimen masses before and after immersion in distilled water, respectively (g); *V*_0_ is the initial specimen volume (cm^3^); and *d*_W_ denotes the density of water (1 g/cm^3^).

#### 2.3.2. Scanning Electron Microscopy (SEM)

Prior to SEM imaging, the samples were coated with a chromium layer (0.2 nm) using a Q150T E plus high-vacuum sputter coater (Quorum Technologies, Laughton, UK). The sputtering process was carried out for 120 s. SEM observations were conducted using a ZEISS SEM 360 microscope (Carl Zeiss Microscopy GmbH, Jena, Germany) operated at an accelerating voltage of 1 kV, with secondary electrons detected in SE2 mode. The tested samples were placed in the electron microscope chamber, and the material surface was analyzed in several micro-regions.

#### 2.3.3. Micro-Computed Tomography

The analyses were performed using a SkyScan 1174 micro-computed tomography scanner (Bruker microCT, Kontich, Belgium) equipped with a 1.3 Mp FW camera. Imaging was performed at a resolution of 11.78 μm. The voltage and current were 50 kV and 800 μA, respectively. The image resolution was 1024 × 1024, the rotation angle was 0.3 (deg), and the number of images per rotation angle was 5. No filter was used during scanning. Tomographic reconstruction was performed in NRecon ver. 1.7.4.2. ROI and 3D model generation in the form of STL extension were performed using CT Analyser ver. 1.23.0.2.

#### 2.3.4. Rheological Testing

Rheological measurements were carried out using a HAAKE Rheostress 6000 rotational rheometer (Thermo Fisher Scientific, Karlsruhe, Germany) in a parallel plate configuration, with an upper plate diameter of 20 mm. This geometry dictated the preparation of cylindrical foam specimens with a matching diameter (20 mm) and an approximate thickness of 3 mm. The lower measuring plate featured a serrated surface to prevent slippage during testing. To ensure consistent contact and reproducible measurement conditions, a constant normal force of 5 N was applied, and the measurement gap was calibrated individually for each specimen based on its exact height.

Prior to testing, all samples were immersed in phosphate-buffered saline (PBS) for 4 h to replicate the conditions of a physiological (wet) environment. All measurements were performed at a controlled temperature of 37 °C to simulate subcutaneous body temperature relevant for biomedical implant applications. Viscoelastic properties were assessed through a creep–recovery test consisting of two sequential phases [35]. In the creep phase, a constant shear stress of *σ*_0_ = 1 kPa was applied for 100 s, followed by a 100 s recovery phase without applied stress. Time-resolved shear strain *γ*(*t*) was continuously recorded and used to calculate creep compliance:

(3) $$J\left(t\right)=\frac{\gamma \left(t\right)}{\sigma_{0}}$$

This compliance function describes the material’s time-dependent deformability, inversely related to its stiffness.

To quantitatively model the mechanical response, the creep data (0–100 s) were fitted using the standard linear solid (Zener) model [36].

(4) $$J\left(t\right)=\frac{1}{E_{1}}+\left(\frac{1}{E_{2}}-\frac{1}{E_{1}}\right)\left(1-e^{-\frac{t}{\tau }}\right)$$

where *E*_1_ = 1/*J*(0) is the instantaneous modulus, *E*_2_ = 1/*J*(∞) is the equilibrium modulus and *τ* is the retardation time, *τ* = *η*/*E_K_*, where *η* and *E_K_* are the viscosity and modulus of the Kelvin–Voigt element, with 1/*E*_2_ = 1/*E*_1_ + 1/*E_K_*.

The recovery phase (100–200 s) was analyzed using a four-parameter Weibull function [37]:

(5) $$J\left(t\right)=J_{v}\cdot e^{{-\left(\frac{t-t_{0}}{\lambda }\right)}^{\beta }}+J_{p}$$

with *J_v_* denoting the reversible (viscoelastic) compliance, *J_p_* the permanent deformation, *λ* the characteristic recovery time, and *β* the shape parameter of the relaxation curve. Here, *t*_0_ = 105 s corresponds to the first data point after unloading.

The resulting shear strain did not exceed 0.92%. As the linear viscoelastic limit was not determined, both models were used as phenomenological descriptions of the creep and recovery curves to compare the two foams under identical conditions.

All curve fitting procedures, including Zener and Weibull models, were performed using OriginPro 2024 (OriginLab Corporation, Northampton, MA, USA) with nonlinear least squares regression. All tests were conducted in five replicates per material (*n* = 5), and results are reported as mean ± standard deviation. Statistical significance was defined at *p* < 0.05.

#### 2.3.5. X-Ray Photoelectron Spectroscopy (XPS)

XPS measurements were performed using a PREVAC multichamber ultra-high-vacuum (UHV) system (PREVAC Sp. z o.o., Rogów, Poland). The setup included a Scienta R4000 hemispherical electron analyzer (VG Scienta AB, Uppsala, Sweden) and a Scienta SAX-100 X-ray source (VG Scienta AB, Uppsala, Sweden) operating with Al Kα radiation (1486.6 eV; bandwidth 0.8 eV), coupled with an XM 650 X-ray monochromator (bandwidth 0.2 eV). Survey spectra were acquired at an analyzer pass energy of 200 eV with an energy step of 750 meV. High-resolution spectra of the C 1s, O 1s, and N 1s regions were recorded using a pass energy of 50 eV and a step size of 100 meV. The analysis chamber maintained a base pressure of 5 × 10^−9^ mbar, which remained below 1 × 10^−8^ mbar throughout data acquisition.

#### 2.3.6. Thermogravimetric Analysis (TGA)

The thermal stability of the PU matrices was evaluated by thermogravimetric analysis using a TGA Q500 instrument (TA Instruments, New Castle, DE, USA). Measurements were carried out under a nitrogen atmosphere, with the temperature increased from 30 to 600 °C at a heating rate of 10 °C/min.

#### 2.3.7. Radiation Sterilization

Gamma irradiation was performed in air at room temperature using a Gamma Chamber GC5000 equipped with a cobalt-60 source (Board of Radiation & Isotope Technology, BRIT, Navi Mumbai, India). The samples were irradiated to an absorbed dose of 25 kGy at a dose rate of approximately 1.2 kGy/h. The absorbed dose was verified using alanine dosimetry.

### 2.4. Preparation of Control and DOX-Loaded Samples

G and G3^gl^G foams were cut into rectangular shapes (10 mm × 10 mm × 5 mm), precisely trimmed and divided into experimental groups: incubation in ethanolic DOX solution (1000 µM), ethanol (solvent control), and untreated control. Samples intended for encapsulation analysis were additionally weighed to determine the initial dry mass.

For drug immobilization and solvent control experiments, individual foam samples were placed in Falcon tubes containing 15 mL of the corresponding solution. Incubation was carried out at room temperature for 24 h, with DOX-containing samples protected from light. Following incubation, the DOX-loaded foams were washed with deionized water to eliminate non-immobilized drug and subsequently vacuum-dried at room temperature to constant mass (3 h). The supernatants obtained after the immobilization step were collected for subsequent DOX quantification. Both the DOX-loaded foams and DOX solutions were kept at 4 °C, protected from light, until analysis.

### 2.5. In Vitro DOX Release Study

The release of DOX from G and G3^gl^G foams was investigated using an Incu-Shaker Mini orbital incubator (Benchmark Scientific, Sayreville, NJ, USA). Drug-loaded specimens were placed in 45 mL of PBS (pH 7.4) and maintained at 37 ± 0.5 °C under orbital shaking (100 rpm) for 100 h. Collected samples were stored at 4 °C for further analysis.

### 2.6. Fluorescence Determination of DOX Concentration

DOX concentration in post-release and post-immobilization solutions was determined using an Infinite M200 PRO microplate reader (TECAN Group Ltd., Männedorf, Switzerland). Measurements were performed in black flat-bottom 96-well plates (100 µL per well) at excitation/emission wavelengths of 480/590 nm. Calibration standards (0–5 µM) were prepared from doxorubicin hydrochloride (DOX·HCl) in PBS and fitted using least-squares linear regression. Fluorescence signals were corrected using the respective blanks. All measurements were performed in triplicate. Post-release samples were analyzed directly. Ethanolic supernatants collected after DOX immobilization and the initial immobilization solution (C_0_ = 1000 µM) were diluted 250-fold with PBS to obtain a concentration within the linear range of the calibration curve. A PBS–ethanol mixture at the same final ratio served as a blank. DOX concentrations in the original solutions were calculated according to the total dilution factor.

### 2.7. Determination of DOX Loading and Encapsulation Efficiency

The amount of DOX immobilized in G and G3^gl^G foams was determined by subtracting the the mass of DOX remaining in the solution after immobilization from its initial mass (*m*_0_). Both quantities were established using fluorescence measurements.

Encapsulation efficiency (*EE*%) and drug loading capacity (*LC*%) were calculated according to Equations (6) and (7).

(6) $$EE(\%)=\frac{m_{load}}{m_{0}}\times 100$$

(7) $$LC(\%)=\frac{m_{load}}{m_{foam}}\times 100$$

where *m*_load_ is the mass of DOX incorporated into the foam and *m*_foam_ is the initial dry mass of the sample.

### 2.8. Cell Culture

SCC-15 human squamous cell carcinoma cells obtained from American Type Culture Collection (ATCC, Manassas, VA, USA) was cultured in DMEM/F12 medium supplemented with 0.4 µg/mL hydrocortisone. Human immortalized keratinocytes (HaCaT) from Cell Line Service (CLS, Eppelheim, Germany) were maintained in DMEM. Both media were enriched with 10% heat-inactivated fetal bovine serum (FBS), 100 U/mL penicillin, and 100 μg/mL streptomycin (all from Corning, NY, USA). Cells were incubated at 37 °C with 5% CO_2_ and 95% humidity. Routine maintenance comprised medium replacement every 2–3 days and passaging at approximately 80% confluence with 0.25% trypsin–0.03% EDTA in sterile Ca^2+^- and Mg^2+^-free PBS. Cultures were routinely checked for morphology under a Nikon TE2000S phase-contrast microscope (Nikon Corporation, Tokyo, Japan), and cell number and viability were determined by trypan blue exclusion with a TC20™ Automated Cell Counter (Bio-Rad, Hercules, CA, USA).

### 2.9. Direct Contact Cytotoxicity Assay

All foam samples intended for cytotoxicity studies were irradiated with 9 MeV accelerated electrons using an “Elektronika 10/10” linear accelerator (NPO TORIJ, Moscow, Russia) at the Institute of Nuclear Chemistry and Technology (INCT, Warsaw, Poland), as described previously [31]. The cytotoxicity of the obtained foams was determinated using a direct contact assay according to [30]. SCC-15 squamous carcinoma cells and HaCaT immortalized keratinocytes were seeded into 6-well plates at a density of 8 × 10^5^ cells per well and allowed to adhere for 24 h at 37 °C in an atmosphere of 5% CO_2_ and 95% humidity. The medium was then aspirated and the wells were rinsed with 1 mL of fresh medium. Sterile foam specimens (10 mm × 10 mm × 5 mm) were placed directly onto the cell monolayers and incubated for 24 h. After specimen removal, the cells were rinsed with PBS and stained for 30 min with 0.2% crystal violet in 2% ethanol. The monolayers were then washed three times with water, imaged, and the reactivity zones were quantified in ImageJ 1.49v (National Institutes of Health, Bethesda, MD, USA); reactivity grades were interpreted according to [38] Bound dye was subsequently released with 10% acetic acid (1 mL per well) by shaking for 10 min at 400 rpm at room temperature, and the absorbance of the resulting solutions was measured at 595 nm against a 450 nm reference on a µQuant™ microplate reader (BioTek Instruments, Winooski, VT, USA). Results were expressed as a percentage of the untreated control. All experiments were performed in triplicate in three independent repetitions.

### 2.10. Extract Cytotoxicity Assay with Neutral Red (NR)

Extracts of the sterilized foams were prepared in complete DMEM/F12 medium in accordance with [39]. Briefly, foams sterilized as described above (four pieces, 10 mm × 10 mm × 5 mm) were fully immersed in 5.33 mL of DMEM/F12 and incubated at 37 °C for 72 h with shaking, giving a surface-area-to-volume ratio of 3 cm^2^/mL. The extracts were then diluted in complete medium to final concentrations of 3.125–100%. HaCaT and SCC-15 cells were seeded into 96-well plates (5 × 10^3^ cells per well in 100 µL of medium) and allowed to adhere for 24 h, after which the medium was removed and replaced with the extracts. Cells were exposed to the extracts for 72 h at 37 °C; cells maintained in native DMEM/F12 medium served as the control. The medium was then replaced with 2% neutral red in complete medium (100 µL per well) and the cells were incubated for a further 1 h. After rinsing with warm PBS, 100 µL per well of fixative (50% ethanol, 49% water, 1% glacial acetic acid) was added and the plates were shaken until the dye was completely released (450 rpm, 15 min, room temperature). Absorbance was measured at 540 and 620 nm on a µQuant™ microplate reader (BioTek Instruments, Winooski, VT, USA) against a blank consisting of fixative without cells. Cell viability was calculated by normalizing the absorbance values to those of the untreated controls, set as 100%.

### 2.11. In Vitro Skin Irritation Testing Using a Reconstructed Human Epidermis Model

The skin irritation test was performed according to a modified MatTek (MatTek Corporation, Ashland, MA, USA) OECD 439 protocol in combination with the MatTek ET-50 procedure using the reconstructed human epidermis (EpiDerm™) model. The EpiDerm™ model consists of normal human epidermal keratinocytes cultured to form a multilayered, differentiated tissue containing basal, spinous, granular, and stratum corneum layers. Samples were applied to the tissue surface for 18 h (±0.5 h), after which cell viability was determined using the MTT assay, which measures mitochondrial enzymatic activity as an indicator of viable cells. The irritancy potential was evaluated based on the relative tissue viability compared to the negative control (PBS-treated tissues). According to OECD 439 criteria, samples were classified as irritant when the relative tissue viability was ≤50%.

### 2.12. Biocompatibility of Tested Foams Against C. elegans

*Caenorhabditis elegans* was used to assess the safety of foams with or without glycidylated dendrimers and (DOX). Wild-type nematodes (N2 strain) were maintained at 20 °C on NGM agar plates seeded with the *Escherichia coli* OP50 strain and synchronized as described earlier [29]. About 20 worms in 50 µL were transferred to each well of a 96-well plate, followed by the addition of 50 µL of the tested extracts from each type of foam. Extracts from foams were prepared in accordance with ISO 10993-12 guidelines as described above on complete S medium [38] and solutions in the range of 12.5–100% were used. Plates were incubated at 20 °C for seven days, during which live and dead nematodes were counted every day using Delta Optical IB-100, inverted microscope (Delta Optical, Nowe Osiny, Poland).

### 2.13. Statistical Analysis

Because the data did not meet the assumption of normality, differences between matrices or extract-treated cells and the untreated control were assessed within each cell line using the non-parametric Kruskal–Wallis test and post-hoc Dunn test with Bonferroni correction. Differences were considered statistically significant at *p* ≤ 0.05. All calculations were performed in Statistica 13 (StatSoft, Tulsa, OK, USA).

## 3. Results and Discussion

### 3.1. Design and Preparation of Hybrid PU-PAMAM G3^gl^ Foams

The development of hybrid PU foams containing hydroxylated PAMAM dendrimers required addressing the limited miscibility of the dendrimer with conventional polyether polyols. PAMAM G3^gl^ exhibits a semi-solid physical state at room temperature, which significantly hinders its direct incorporation into the polyol phase. Consequently, the formation of a homogeneous hybrid polyol system required the introduction of an additional component facilitating the dispersion of the dendrimer. Therefore, glycerol was introduced as a compatibilizing and fluidizing agent. The presence of glycerol enabled the formation of a stable ternary polyol system composed of PAMAM G3^gl^, glycerol, and polyether triol. Such an approach allowed efficient incorporation of the dendrimer into the PU precursor mixture.

The influence of the polyol structure on the formation of the hybrid system was also evaluated. Initial experiments performed using Rokopol G441 resulted in incomplete homogenization and irregular foam morphology. Replacement of this polyol with Rokopol G1000 significantly improved blend uniformity and resulted in foams with a more regular cellular structure. This observation indicates that the architecture and molecular weight of the polyether polyol strongly influence the formation of the hybrid PU network.

In order to determine the optimal dendrimer content, formulations containing 20, 30, and 40 wt% PAMAM G3^gl^ were investigated. Increasing the dendrimer fraction required maintaining an equivalent amount of glycerol to ensure sufficient miscibility within the hybrid polyol system. As a consequence, the overall hydroxyl functionality of the system increased, leading to a higher crosslink density of the resulting PU network. Foams containing more than 20 wt% dendrimer exhibited increased brittleness and a disturbed porous morphology, indicating excessive crosslinking of the polymer network. Based on these observations, the optimal polyol composition was determined as 20 wt% PAMAM G3^gl^, 20 wt% glycerol, and 60 wt% polyether triol (G1000). This composition produced semi-rigid PU foams characterized by a relatively regular cellular morphology. Reference foams prepared without the dendrimer exhibited typical structural characteristics of conventional PU foams, confirming that the observed structural changes were associated with the incorporation of the PAMAM-based component.

### 3.2. XPS Analysis of Hydroxylated Dendrimer Incorporation into Polyurethane Foams

To evaluate the surface chemical composition and to verify the incorporation of the hydroxylated PAMAM G3 dendrimer into the PU matrix, XPS analysis was performed. XPS analysis revealed the presence of carbon, oxygen, and nitrogen as the main surface elements in both the dendrimer-modified foam and the reference foam, which is consistent with the chemical structure of PU materials containing urethane linkages. Additional data are presented in the Supporting Information (Figures S1 and S2, Table S1).

**Figure 1:**
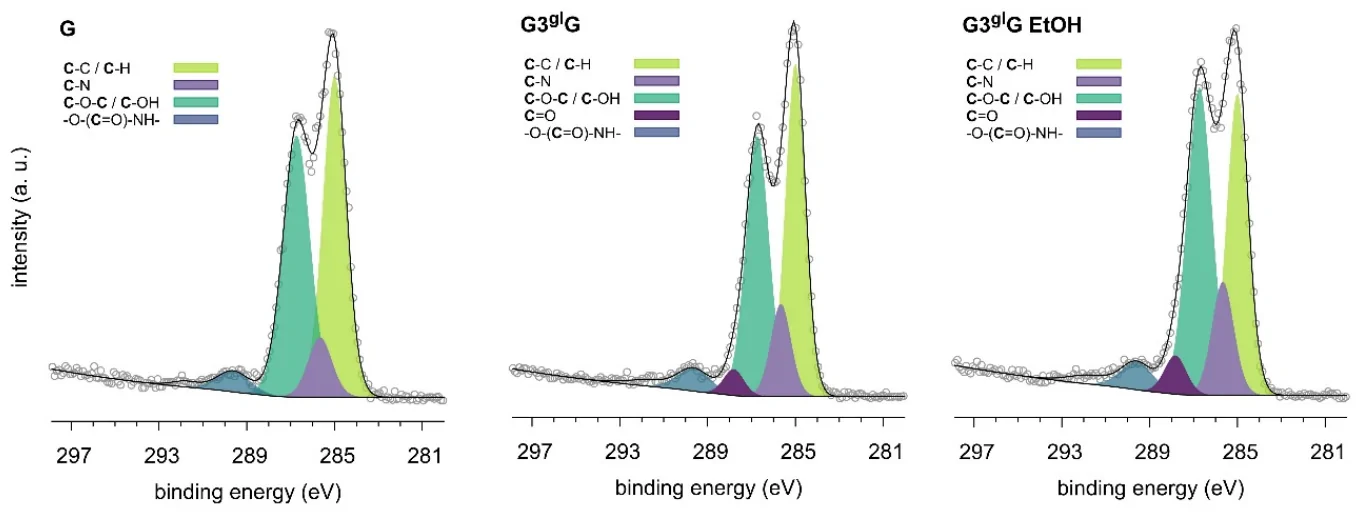
High-resolution C 1s spectra of reference (G) and dendrimer-modified (G3^gl^G) polyurethane foams before and after ethanol treatment. The additional component at ~287.8 eV observed for G3^gl^G is attributed to the carbonyl groups of the PAMAM dendrimer. The open circles represent the experimental data, while the black solid line represents the overall fitted spectrum.

In comparison to the reference foam G, dendrimer-modified samples G3^gl^G exhibited a higher nitrogen content (4.50 vs. 3.90 at.%), which is consistent with the nitrogen-rich structure of PAMAM G3. High-resolution spectra of the C 1s region revealed an additional component at ~287.8 eV in G3^gl^G foam, which can be attributed to carbonyl groups of PAMAM G3 dendrimer (Figure 1). Quantitative analysis of G3^gl^G before and after ethanol extraction showed a decrease in surface nitrogen content from 4.5 to 3.8 at.%, while the relative contribution of the C 1s component at approximately 287.8 eV changed from 3.4% to 4.6%. The principal N 1s component was observed at approximately 400.5 eV in both samples. These results confirm indicate the persistence of the corresponding surface chemical features after ethanol treatment (Table S1).

**Scheme 1:**
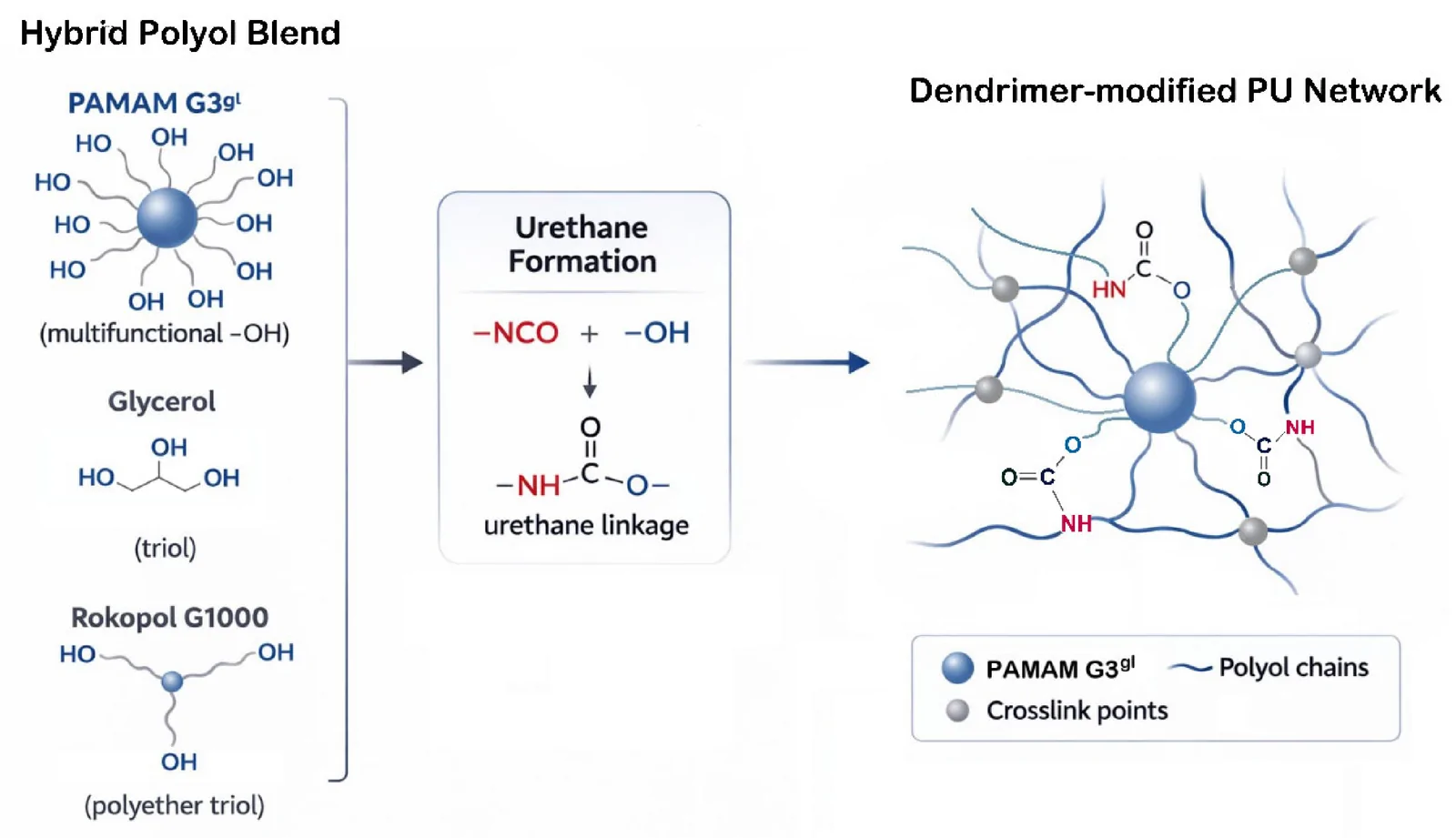
Schematic representation of the proposed incorporation of hydroxylated PAMAM G3 dendrimer into the polyurethane network.

The persistence of this additional C=O component in the C 1s spectra of dendrimer-containing foams after ethanol extraction, despite a moderate decrease in surface nitrogen content, indicates that hydroxylated PAMAM G3remains associated with the PU network (Scheme 1). Furthermore, the retention of the nitrogen signal together with the additional carbonyl component after solvent extraction supports the stable incorporation of the dendrimer into the polyurethane structure. This observation are consistent with the proposed formation of covalent urethane linkages between hydroxyl groups present on the PAMAM G3^gl^ surface and isocyanate groups of pMDI during the foaming reaction.

### 3.3. Morphology and Related Physicochemical Properties of PAMAM G3 Polyhydroxylated Dendrimer-Based Foam

**Figure 2:**
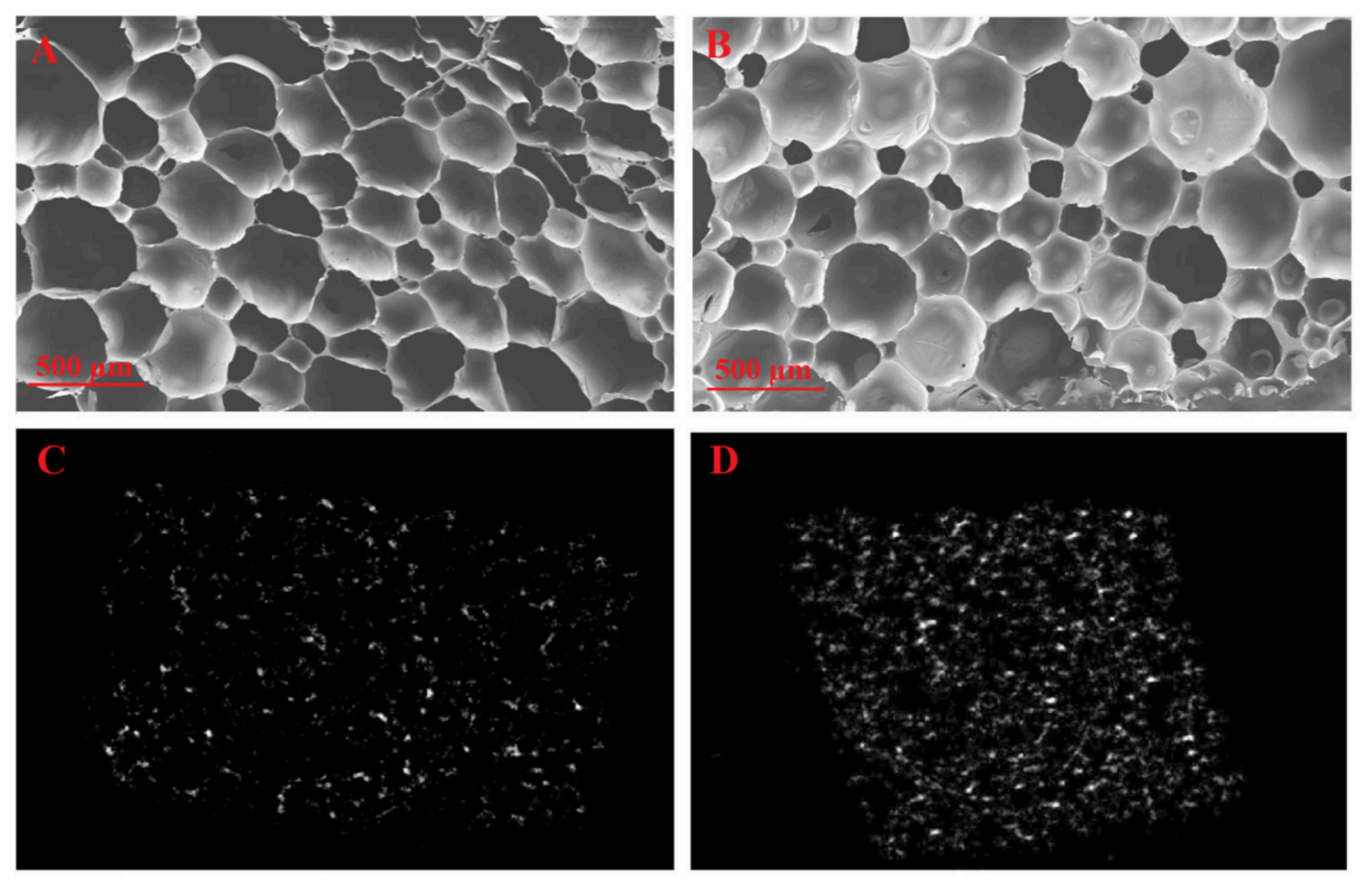
Morphological characterization of porous PU-PAMAM G3^gl^ and reference foams. SEM micrographs of (**A**) reference material (G) and (**B**) dendrimer-modified material (G3^gl^G) showing well-defined cellular architectures. µCT cross-sections of (**C**) G and (**D**) G3^gl^G confirming homogeneous internal porous structure without visible macroscopic defects. No pronounced structural collapse or large-scale discontinuities were observed upon dendrimer incorporation. The µCT cross-sections (**C**,**D**) represent approximate fields of view of 10 mm.× 10 mm and 12 mm × 12 mm, respectively.

Scanning electron microscopy (SEM) revealed that both the reference material (G) and the dendrimer-modified material (G3^gl^G) exhibited well-developed cellular architecture (Figure 2A,B).

The cells were generally spherical with continuous walls, and no visible cracks or structural fractures were observed in the examined regions. The incorporation of PAMAM G3^gl^ did not disrupt the foaming process, indicating that the hybrid polyol mixture enabled uniform cell growth during the foaming process. Quantitative analysis of the SEM micrographs revealed comparable mean pore diameters for material G (314.5 ± 103.7 µm) and G3^gl^G (301.6 ± 92.3 µm). Although the mean values were similar, the reference material exhibited a wider pore size distribution, including occasional larger cells. In contrast, the dendrimer-modified material exhibited a slightly narrower distribution range, suggesting a more uniform cellular structure. These observations indicate that the incorporation of PAMAM G3^gl^ does not significantly alter the characteristic pore size scale but may impact structural uniformity. Microcomputed tomography (µCT) cross-sections of both materials (Figure 2C,D) confirmed the internal cellular organization throughout the bulk of the material. The reconstructed images revealed no macroscopic voids, depressions, or structural discontinuities. Within the resolution limits of this technique, dendrimer incorporation did not introduce observable large-scale morphological defects.

Despite comparable cellular morphology, dendrimer incorporation affected the macroscopic physicochemical properties (Table 2).

The apparent density of G3^gl^G (53.7 kg/m^3^) was lower than that of the reference material G (63.0 kg/m^3^), indicating a lighter cellular structure. This difference may be related to subtle changes in the gas expansion behavior or viscoelastic properties of the matrix during foaming in the presence of the hydroxyl-functional dendrimer.

Water absorption increased with immersion time in both materials. However, G3^gl^G consistently showed higher absorption values (3.32%, 5.64%, and 9.05% after 5 min, 3 h, and 24 h, respectively) compared to G (3.04%, 4.56%, and 7.11%, respectively). The increased water absorption may be associated with the increased polarity introduced by the dendrimer component. Overall, the results show that PAMAM G3^gl^ can be incorporated into a PU matrix without compromising the cellular architecture, while moderately modifying the density and water absorption properties, relevant for subsequent drug immobilization studies.

### 3.4. Creep–Recovery Behavior of PU-PAMAM G3^gl^ Matrix

**Figure 3:**
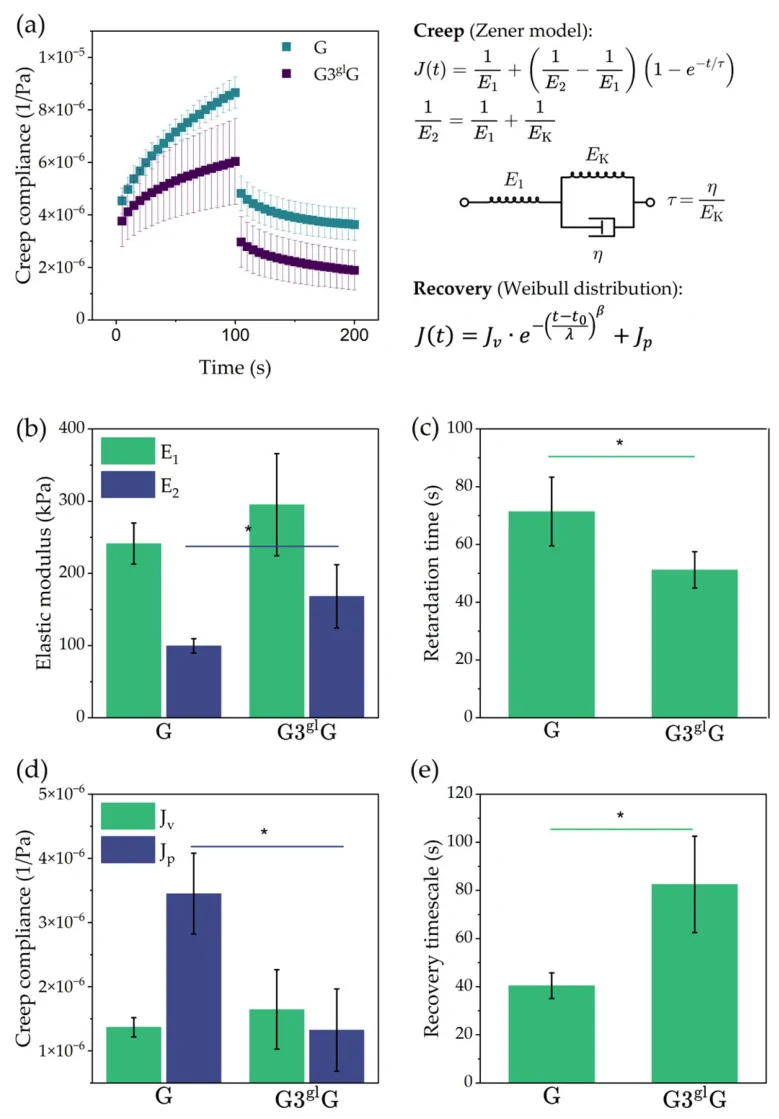
Creep–recovery analysis of polyurethane foams. (**a**) Creep compliance at 1 kPa (100 s) and recovery (100 s) for G and G3^gl^G foams, with Zener and Weibull model fits. (**b**) Elastic moduli E_1_ and E_2_. (**c**) Retardation time τ. (**d**) Reversible (J_v_) and irreversible (J_p_) compliance. (**e**) Recovery time λ. Error bars: SD (*n* = 5); * *p* < 0.05.

Creep–recovery tests were performed to characterize the time-dependent mechanical response of the PU matrices. A constant shear stress of 1 kPa was applied for 100 s to induce deformation, followed by a 100 s recovery period. The creep phase was analyzed using the Zener model to extract instantaneous (E_1_) and equilibrium (E_2_) elastic moduli. The recovery phase was fitted with a four-parameter Weibull function to quantify reversible strain (J_v_), permanent deformation (J_p_), characteristic recovery time (λ), and shape parameter (β). Figure 3a presents the average creep–recovery curves for unmodified G foam (based on Rokopol G1000) and dendrimer-modified G3^gl^G.

During the creep phase, unmodified G foam exhibited higher compliance, indicating lower resistance to deformation. In contrast, G3^gl^G showed reduced strain accumulation, suggesting that dendrimer incorporation enhances creep resistance. During the recovery stage, exhibited partial strain recovery, indicative of viscoelastic behavior, with G3^gl^G showing more pronounced recovery and lower permanent deformation.

The Zener model parameters reveal distinct effects of dendrimer modification (Figure 3). The instantaneous modulus E_1_ remained similar in both groups (241 ± 29 kPa for G vs. 295 ± 71 kPa for G3^gl^G), indicating that short-term elastic response was unaffected by modification. However, the equilibrium modulus E_2_ increased substantially by 69% in G3^gl^G (168 ± 44 kPa vs. 100 ± 10 kPa), reflecting improved resistance to time-dependent deformation. The retardation time τ decreased from 71 ± 12 s in G to 51 ± 6 s in G3^gl^G (Figure 3c), indicating that the modified foam approached its equilibrium compliance faster.

Analysis of the recovery phase revealed significant improvements in structural reversibility (Figure 3). The reversible viscoelastic compliance J_v_ increased slightly after modification (from 1.37 × 10^−6^ to 1.65 × 10^−6^ Pa^−1^), while the irreversible component J_p_ decreased by approximately 62% in G3^gl^G (from 3.45 × 10^−6^ to 1.32 × 10^−6^ Pa^−1^). This substantial reduction in permanent deformation confirms enhanced structural recovery. The characteristic recovery time λ increased from 40 ± 5 s in unmodified G to 83 ± 20 s in G3^gl^G (Figure 3e), indicating slower but more complete strain reversal. This extended recovery, combined with reduced J_p_, suggests improved dimensional stability under repetitive loading. Because λ of G3^gl^G is comparable to the 100 s duration of the recovery stage, its value depends on the fitting window and should be regarded as an estimate; the reduction of Jp was independent of the window. The shape parameter β showed no significant difference between samples (0.81 ± 0.07 for G vs. 0.74 ± 0.05 for G3^gl^G), indicating that dendrimer modification affected the extent and kinetics of recovery without altering the fundamental relaxation mechanism. The measurements were performed at a single stress level; the parameters therefore characterize the two foams under identical conditions, and their absolute values may depend on the applied stress.

### 3.5. Effect of Radiation Sterilization on Thermal Stability of PU–PAMAM G3^gl^ Materials

The thermal behavior of PU–PAMAM G3^gl^ foam following radiation sterilization was investigated by thermogravimetric analysis (TGA/DTG) under a nitrogen atmosphere (Figure S3). Samples were exposed to either gamma radiation (Co-60) or an electron beam (10 MeV), with an absorbed dose of 25 kGy in both cases.

The onset of thermal degradation, represented by T_5_% and T_10_%, remained nearly unchanged after irradiation (Table 3).

This indicates that exposure to ionizing radiation did not induce early-stage polymer degradation or chain scission detectable by TGA. More pronounced differences were observed at higher levels of mass loss. The temperature corresponding to 50% weight loss (T_50_%) increased from 348.8 °C for the non-irradiated material to 362.2 °C after gamma irradiation and to 355.1 °C after electron-beam treatment. Similarly, the main DTG degradation peak (T_2_) shifted toward higher temperature for the gamma-irradiated sample (330.1 °C → 339.2 °C), whereas only minor changes were observed for the electron-beam-treated material. A substantial increase in residual mass was detected after irradiation (4.6% for control vs. 14.8% for gamma and 12.6% for electron beam). This behavior suggests enhanced formation of thermally stable carbonaceous structures during decomposition. Such an effect may be associated with radiation-induced crosslinking or structural rearrangements within the PU–dendrimer network. Overall, the results indicate that sterilization at a dose of 25 kGy does not negatively impact the thermal degradation profile of the PU-PAMAM G3^gl^ matrix. Gamma irradiation even resulted in a slight improvement in thermal stability, as reflected by the shift of T_50_% and T_2_ toward higher temperatures.

### 3.6. Drug Loading and Surface Analysis of DOX Immobilization

Quantification of doxorubicin (DOX) loading was performed by fluorescence mass balance analysis of the post-incubation supernatants. Results of DOX loading are summarized in Table 4.

**Figure 4:**
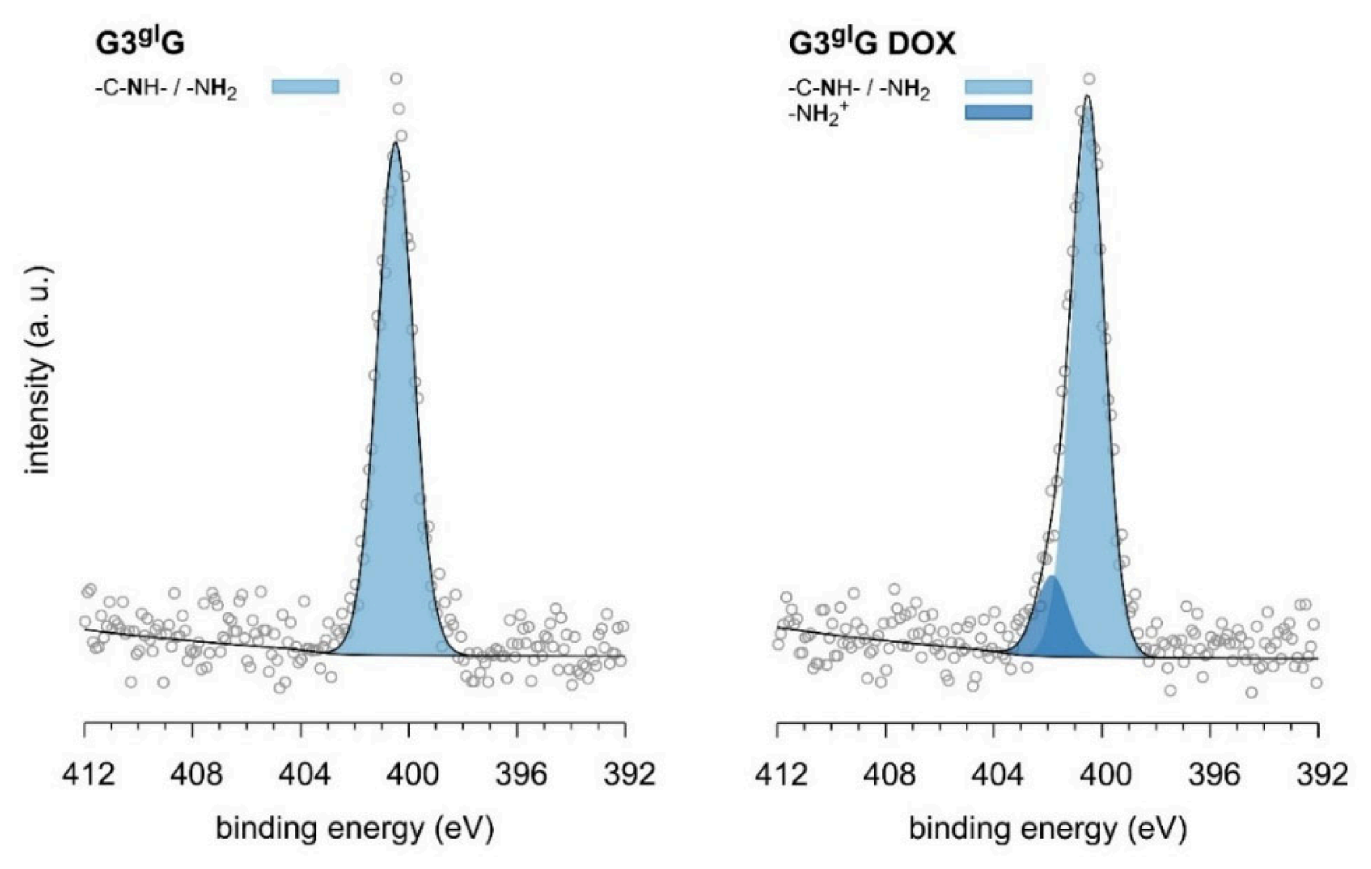
XPS analysis of DOX-loaded PU-PAMAM G3^gl^ matrix after DOX immobilization. High-resolution N 1s spectra demonstrating the additional protonated amine component (–NH_3_^+^) characteristic of doxorubicin hydrochloride. The open circles represent the experimental data, while the black solid line represents the overall fitted spectrum.

At an initial DOX concentration of 1000 µM, the reference foam (G) showed an encapsulation efficiency (EE) of 29.7%, corresponding to a loading capacity (LC) of 10.3 wt%. Incorporation of hydroxylated PAMAM G3^gl^ increased the EE to 34.9% and the LC to 12.0 wt%, indicating moderately increased drug retention in the dendrimer-modified PU matrix. This difference suggests more favorable drug–matrix interactions in G3^gl^G under the investigated loading conditions, potentially associated with the increased network polarity and the presence of functional groups within the dendrimer structure. X-ray photoelectron spectroscopy (XPS) provided additional confirmation of DOX immobilization (Figure 4).

High-resolution N 1s spectra of DOX-immobilized samples revealed an additional component with a higher binding energy (~401–403 eV), attributed to the protonated amine groups (–NH_3_^+^) characteristic of doxorubicin hydrochloride. This component was absent in the reference foams without DOX, confirming the presence of DOX on the material surface. It is important to emphasize that fluorescence-based mass balance reflects the total drug content in the entire foam volume, whereas XPS examines only the outermost, nanometric layer of the material. Therefore, XPS results provide qualitative confirmation of the presence of surface-bound DOX rather than a quantitative measurement of the total drug loading. Encapsulation efficiency (EE) and loading content (LC) obtained in this study for G3^gl^G matrix (EE ≈ 35%, LC ≈ 12%) are within the range reported for PAMAM-based drug carriers [25].

The higher DOX immobilization observed for G3^gl^G compared with the reference foam may be associated with additional drug–matrix interactions introduced by the PAMAM dendrimer within the polyurethane network.

### 3.7. In Vitro Release

**Figure 5:**
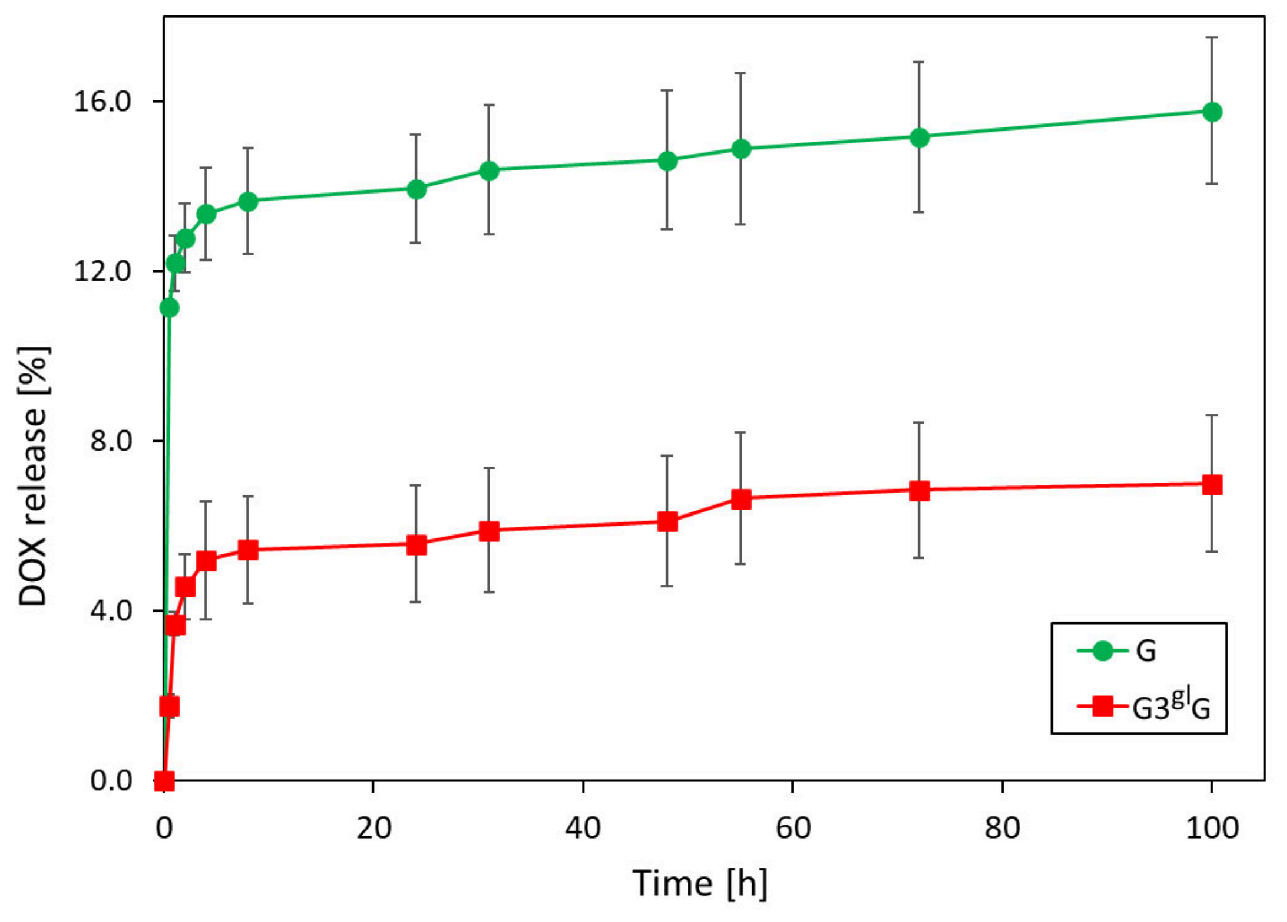
Cumulative release profiles of doxorubicin from PU and PU-PAMAM G3^gl^ matrices in PBS (pH 7.4, 37 °C) over 100 h.

The cumulative release profiles were biphasic for both matrices, comprising a rapid initial phase completed within the first hours of incubation and a subsequent slow, quasi-linear phase extending to 100 h. Cumulative DOX release reached 15.76% for the reference G and 6.97% for the dendrimer-modified G3^gl^G foams under identical conditions (PBS, pH 7.4, 37 °C) after 100 h. The G reference system exhibited a higher initial release within the first hour, followed by gradual stabilization. In contrast, the G3^gl^G matrix demonstrated reduced early-stage burst release and a slower overall release profile, indicating enhanced drug retention in the dendrimer-containing network (Figure 5).

Both drug delivery systems differed markedly in the relative contribution of the two phases. For the unmodified PU matrix, 11.1% of the total payload was released within the first 30 min, corresponding to approximately 71.1% of the entire amount liberated over 100 h. Release reached 13.6% at 8 h and thereafter increased by only 2.2 percentage points over the remaining 92 h, attaining a final value of 15.8%. The PU–PAMAM G3^gl^ matrix released 1.8% over the same initial interval, that is roughly one quarter of its 100 h total, followed by a progressive increase to 5.4% at 8 h and 7.0% at 100 h. Accordingly, the fraction of drug delivered during the sustained phase was proportionally higher for the modified matrix (22% versus 13% of the respective totals), and its profile had not reached a plateau at the end of the observation window. The suppression of burst release in the dendrimer-modified matrix is consistent with a change in the population of accessible drug. Burst release from porous matrices originates predominantly from drug adsorbed at or near the pore surface, which is exposed to the dissolution medium without a diffusional barrier. In the unmodified foam, DOX loading relies on non-specific partitioning into the polyether-based network and physisorption on pore walls, and both populations are readily desorbed upon hydration. Incorporation of hydroxylated PAMAM G3 introduces a second, chemically distinct binding population: the interior amide carbonyls and tertiary amines of the dendrimer, capable of hydrogen bonding with the protonated daunosamine amine of DOX. The FTIR spectra of the G4.0-DOX complexes confirm the interactions between the components [23]. Release from these sites requires dissociation prior to diffusion, which converts part of the immediately available fraction into a kinetically retarded reservoir. This interpretation is supported by the concurrent increase in encapsulation efficiency (34.9% versus 29.7%), indicating that the additional drug is accommodated at sites that are not simply surface-adsorbed, and by the XPS detection of protonated amine contributions in the N 1s region.

The unmodified foam therefore behaves as a short-acting depot that is largely exhausted within the first day, while the modified foam preserves a reservoir approximately 30% larger together with a release phase that remains active at the end of the experiment. The two profiles are not better and worse versions of the same behavior; they suit different clinical situations. A release pattern dominated by an initial bolus, as in the reference foam, matches settings in which a high instantaneous concentration is the therapeutic objective and the implant is removed or replaced shortly afterwards. The profile obtained for the dendrimer-modified foam is instead suited to the post-resection cavity, where the therapeutic target is the population of residual tumor cells at the margin and the relevant exposure parameter is the time above the cytotoxic threshold rather than the peak concentration. In this setting a pronounced burst is doubly disadvantageous: it exhausts the depot before the period of highest recurrence risk, and the transient peak is dissipated by convective and lymphatic clearance without contributing to sustained exposure. Retaining the payload, as the PU–PAMAM G3^gl^ matrix does, converts the implant from a bolus into a reservoir. The additional argument in favor of the modified matrix is architectural rather than kinetic: unlike injectable or gel-based depots, the foam is retained at the implantation site, can be cut to fill an irregular cavity, and maintains the residual drug in immediate contact with the tissue margin throughout the release period.

### 3.8. Direct Contact Cytotoxicity Assay

Foams are a particular type of material owing to their flexibility, longevity, and resilience [9]. They offer several advantages when introduced into body cavities, the most important being their substantial post-application expansion, which enables closer contact between the drug and the mucosal surface [39]. Such materials have to therefore be biocompatible, that is, able to perform their intended function while eliciting an appropriate host response [40]. To assess the properties of the matrices, a direct cytotoxicity assay was performed according to ISO 10993-5. Foam samples were placed onto confluent monolayers of HaCaT or SCC-15 cells, and after 24 h incubation viability was assessed using crystal violet staining.

**Figure 6:**
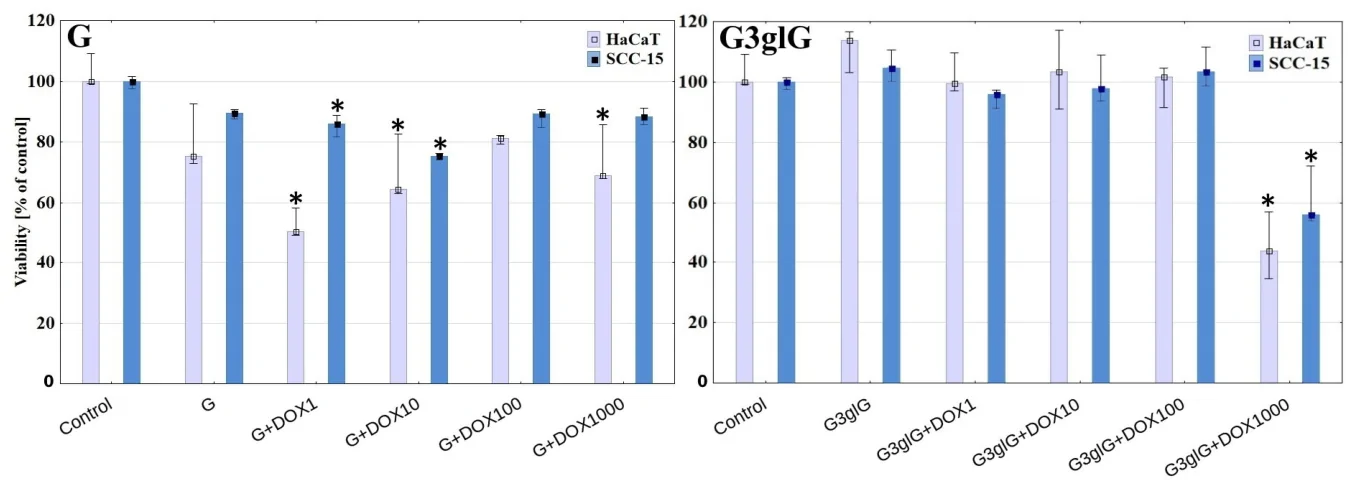
Viability of confluent cultures of human immortalized keratinocytes (HaCaT) and human squamous cell carcinoma cells (SCC-15) after 24 h of exposure to the foam samples, determined by the direct contact cytotoxicity assay. Data are presented as medians of nine measurements from three independent experiments; whiskers denote the lower (25%) and upper (75%) quartiles. Asterisks * indicate statistically significant differences from the untreated control (*p* < 0.05, Kruskal–Wallis test with post hoc Dunn test).

The PU G foam without dendrimers, G + DOX (1, 10, 100, or 1000 μM DOX encapsulated in G as described above), G3^gl^G with/or without DOX containing glycidylated dendrimers were used. G and G3^gl^G foams were highly biocompatible against both used cell lines. Foams without dendrimers, containing DOX showed no clear dose-response. Viability of HaCaT cells decreased by up to 50% at the lowest DOX concentration (G + DOX1 1 μM), whereas in SCC-15 cells the lowest viability was about 75% after G + DOX10 incubation. However, keratinocytes were more sensitive to foams without dendrimers. In contrast, foams containing dendrimers exhibited higher biocompatibility than G samples, even in matrices without the drug. Significant reduction in HaCaT and SCC-15 cell viability was observed only for the samples containing the highest DOX concentration (G3^gl^G + DOX 1000 μM; 57% and 43%, respectively), while no significant changes in cell viability were observed after incubation with other dendrimer- and drug-containing samples (Figure 6).

**Figure 7:**
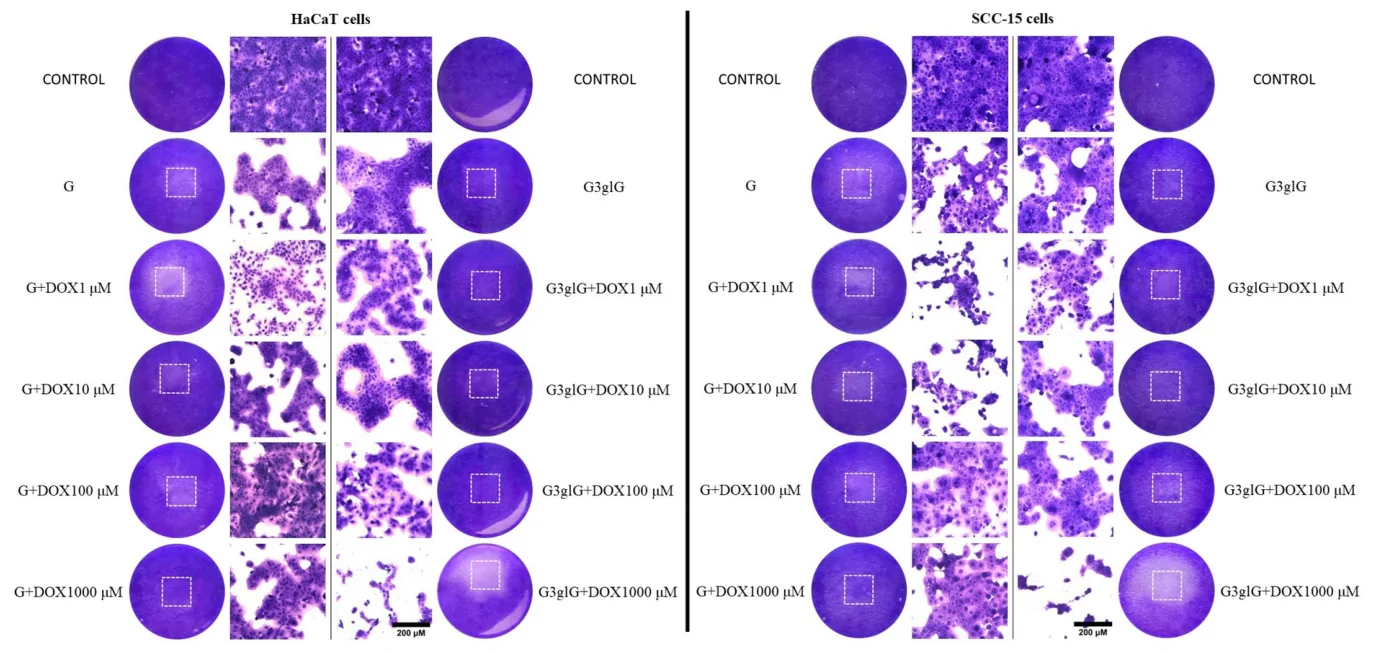
Human immortalized keratinocytes (HaCaT) and human squamous cell carcinoma cells (SCC-15) stained with crystal violet after 24 h of exposure to the tested foams. The circular images show the well bottoms with stained cells, and the white squares mark the areas covered by the specimens. The middle rows show representative cell morphology from the central parts of the reactivity zones.

Images of cells collected after crystal violet staining confirmed these conclusions. According to the reactivity grades (see ISO 10993-5) the G and G3^gl^G matrices indicated only the first, slight degree of reactivity, in which some malformed or degenerated cells under the specimen are seen. Interestingly, the tested cells responded similarly to the remaining matrices, including those containing high DOX amount. Only G3^gl^G + DOX1000 caused grade 3 (moderate) cell damage in both lines, characterized by damage outside the sample-covered area up to 1 cm. This was similar to the G + DOX1 response in HaCaT cells (Figure 7).

### 3.9. Extract Cytotoxicity Assay with Neutral Red (NR)

**Figure 8:**
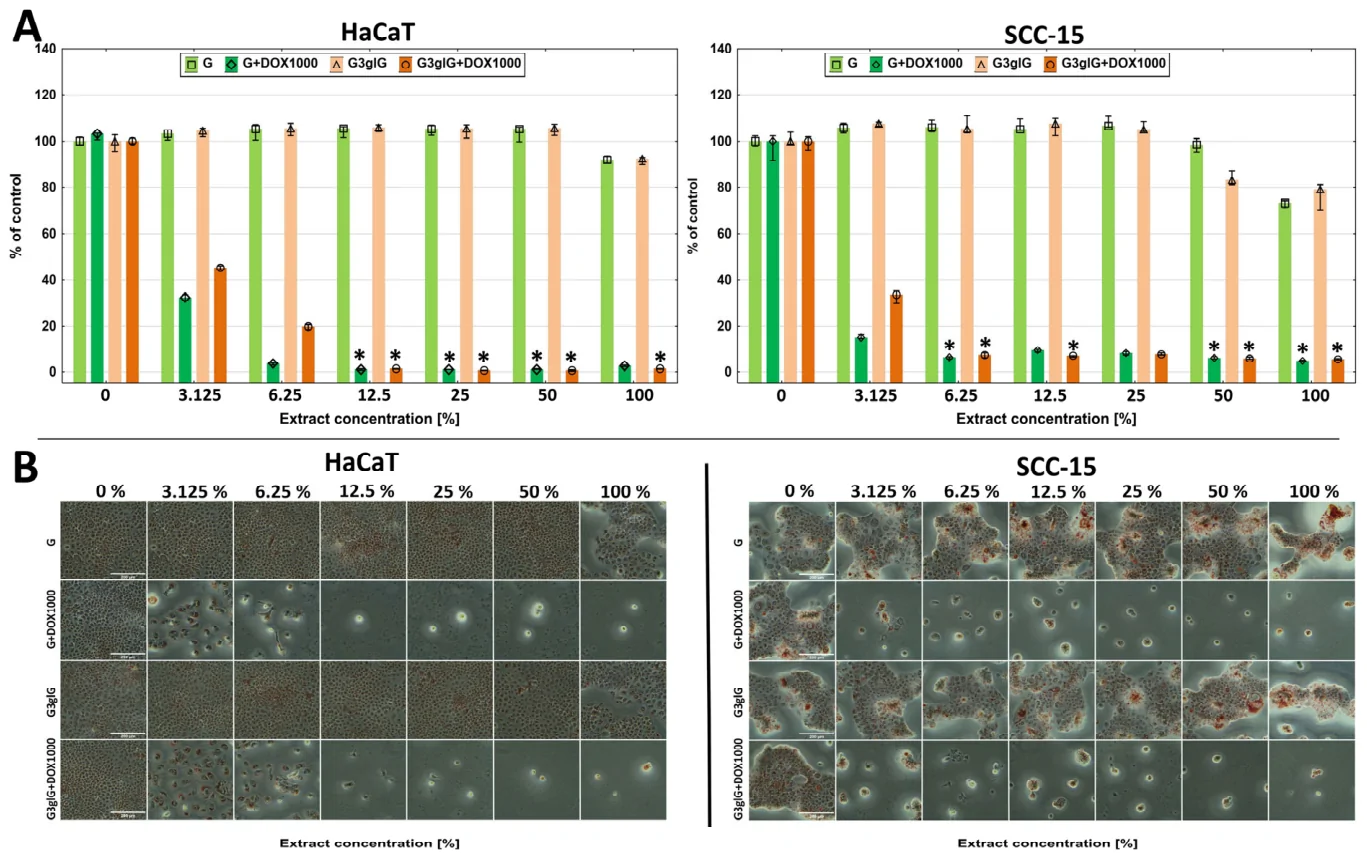
(**A**) Response of HaCaT and SCC-15 cells to extracts from the tested matrices after 72 h incubation, assessed with the NR assay. Data are presented as percentages relative to the untreated control (control = 100% viability). The lower (25%) and upper (75%) quartile ranges are presented as whiskers. Asterisk * indicates statistically significant differences between non-treated control and samples (*p* < 0.05, Kruskal–Wallis test and post-hoc Dunn test). (**B**) Images of studied cells collected after staining with neutral red (NR). Red signal show NR accumulated with lysosomes.

The direct extract assay is considered more sensitive than the indirect assay because the cells come into contact with the tested material. Nevertheless, the extraction method is more suitable for materials that release soluble substances [41]. The direct contact assay is performed on a layer of fully confluent cells. Therefore, it is impossible to study the effect of the material on the proliferation and differentiation processes. The direct contact assay results show that DOX released from the matrices had no effect on the tested cells, despite being highly toxic and having documented antiproliferative effects at nanomolar concentrations [42]. The extract test demonstrated that the G polyurethane matrix and the G3^gl^G containing the dendrimer were highly biocompatible and did not cause a significant decrease in cell viability (Figure 8).

This confirms the stability of the obtained matrices and the lack of leaching of their fragments or unreacted substrates. At the same time, DOX release from both types of matrices caused a drastic decrease in cell viability even at an extract concentration of 3.125%. This decrease was noticeably greater in SCC-15 cancer cells, where cell viability dropped below 20 and 40% (G + DOX1000 and G3^gl^G + DOX1000, respectively). In the case of HaCaT cells, viability at 3.125% extract concentrations was approximately 27 and 45% (G + DOX1000 and G3^gl^G + DOX1000, respectively). At higher concentrations, viability dropped significantly below 10%. The activity of DOX released from G + DOX at concentrations of 3.125 and 6.25 µM was stronger than that of DOX released from G3^gl^G, which aligns perfectly with the DOX release profile of the tested foams.

The earlier studies of free DOX cytotoxicity after 72 h showed that SCC-15 and HaCaT cell viability decreased to zero at 0.625 and 1.25 µM concentration, respectively [31]. The same effect was obtained at 12.5 and 6.25% extracts of DOX released from G and G3^gl^G matrices. This proved effective anticancer action of DOX released from matrices at similar amount. Moreover, it is worth mentioning that the biological activity of both foams—with or without DOX—was slightly stronger against cancer cells than against HaCaT keratinocytes.

### 3.10. In Vitro Skin Irritation Test on Reconstructed Human Epidermis Model

A study conducted on a human epidermis model demonstrated that neither of the tested matrices G and G3^gl^G caused epidermal irritation upon contact with human epidermis. The MTT assay showed that, following 18 h of contact, cell viability exceeded 90% in both cases; therefore, the tested materials should be considered safe and non-irritating (Table 5).

### 3.11. In Vivo Biocompatibility of Tested Foams Against C. elegans

*Caenorhabditis elegans* is a well-established whole-animal model for studying human disease: an estimated 42% of human disease-associated genes have a nematode ortholog [43], while other analyses indicate that its genome contains homologs of roughly two-thirds of all human disease genes [44]. In contrast to cell culture, *C. elegans* assays yield whole-organism data from an animal with functional, metabolically active digestive, reproductive, endocrine, sensory and neuromuscular systems, and toxicity rankings obtained in the nematode have repeatedly predicted rat LD_50_ rankings as well as mouse data do [45]. Concordance with mammalian responses has been demonstrated for aging, aneuploidy and germ cell genome abnormalities, growth and development, LD50 prediction and neurotoxicity [46]. Importantly, findings from this model can translate towards the clinic, as drug screens in *C. elegans* have yielded compounds that suppress disease phenotypes, one of which has progressed to clinical trials [47]. Moreover *C. elegans* has become an important model for the preliminary assessment of biological effects of nanomaterials, further highlighting its relevance in toxicology research [48,49]. Therefore, we selected this organism to obtain an initial in vivo insight into the safety of the proposed foam systems.

**Figure 9:**
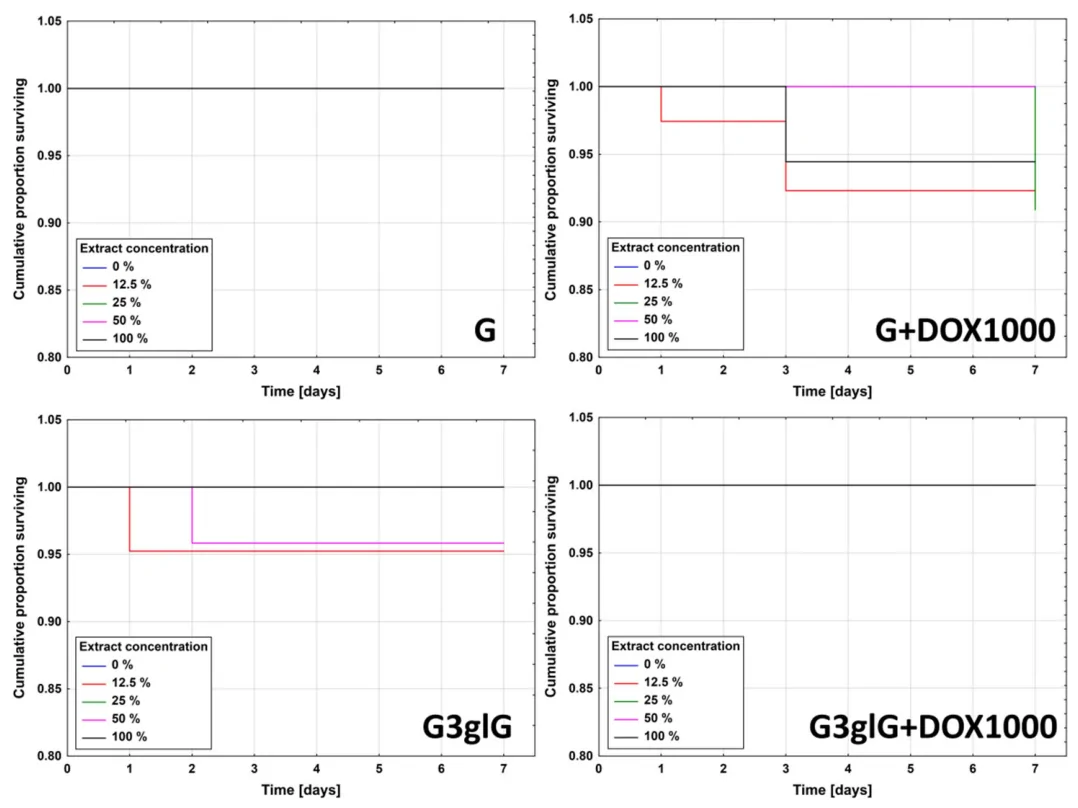
Effect of native G and G3^gl^G matrices and their analogs containing encapsulated doxorubicin (+DOX1000) on the viability of the nematode *C. elegans* after seven days of incubation. Nematode viability is presented using the Kaplan-Meier estimator.

After 7 days, *C. elegans* incubated with extracts of G or G3^gl^G matrices indicated no significant changes in viability. The percentage of surviving individuals was over 95%. Also extracts of matrices with encapsulated DOX (1000 µM concentration of DOX solution) did not cause a viability decrease greater than 8% (Figure 9).

**Figure 10:**
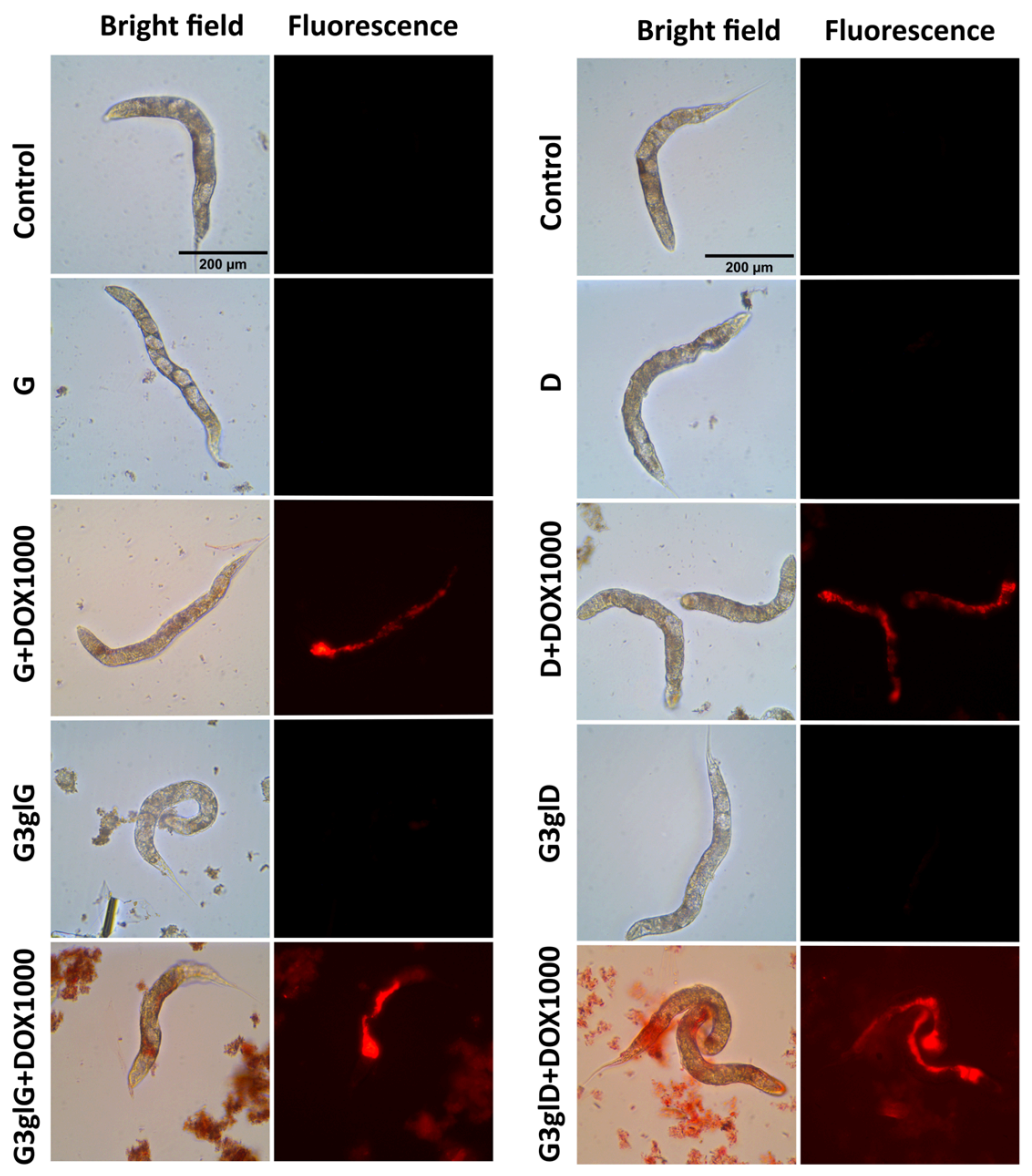
Microscopic images of *C. elegans* collected on day 7 of incubation with foam extracts, showing nematode morphology and DOX accumulation within their bodies (red signal).

Furthermore, fluorescence microscopy observations revealed that doxorubicin released from the matrices penetrated the *C. elegans* organism. Red signal from accumulated DOX was observed along the entire length of the digestive tract but mainly in the pharynx and intestine region which indicates deposition of doxorubicin. Similar observation was described by Balkrishna et al. (2025) [50] in region of pharynx and intestine after 1 day incubation with DOX. It is worth mentioning that the accumulation of DOX released from G3^gl^G matrix was higher than from G (Figure 10).

Balkrishna et al. also showed that DOX after 24 h incubation was not toxic against *C. elegans* up to 5 µM concentration. DOX did not reduce the nematode’s viability, as *C. elegans* is a eutelic species with a constant number of somatic cells, and cell division occurs only in gonads [51]. Doxorubicin is generally considered an antiproliferative drug rather than an acute toxicant [52] therefore did not exert a toxic effect on the studied model organism.

## 4. Conclusions

Hydroxyl-functionalized PAMAM G3 was successfully integrated into polyurethane foams as a reactive polyol component, forming a hybrid network without disrupting porous morphology. Compared with the reference material, the modified foam exhibited improved structural (morphology), physical (increased water absorption) and viscoelastic properties (increased durability and better shape recovery). Moreover, it can be sterilized using gamma radiation or an electron beam and exhibits higher values of drug loading capacity and encapsulation efficiency. It is characterized by gradual, sustained drug release and a reduced initial burst release effect. Biological studies have demonstrated that both studied matrices are highly biocompatible—both in vivo and in vitro—do not cause epidermal irritation, and effectively release doxorubicin, which destroys squamous cell carcinoma SCC-15 cells. Therefore, the use of polyurethane matrices containing hydroxylated PAMAM dendrimers as drug delivery systems for local therapy is worth considering.

## Figures and Tables

**Table 1:** The composition of PU foams based on glycidylated PAMAM dendrimer (G3^gl^), glycerol (Gly), polyether polyol (G1000) and the corresponding foaming parameters.

Foam Marking	Substrate Ratio G3^gl^:Gly:G1000 [wt%]	Substrates							Foaming Process		
		G3^gl^ [g]	G1000 [g]	Gly [g]	pMDI [g]	H_2_O [g]	Silicone [g]	TEA [g]	Time of Creaming [s]	Time of Expanding [s]	Time of Drying [s]
G3^gl^G	20:20:60	0.8	2.4	0.8	5.8	0.16	0.16	0.034	80	195	3120
G	0:20:80	-	3.2	0.8	5.6	0.16	0.16	0.034	110	216	4800

**Table 2:** Apparent density and water absorption of G3^gl^G1000 and the reference foams G1000.

Foam Sample	Apparent Density [kg/m^3^]	Water Absorption [%]		
		After 5 min	After 3 h	After 24 h
G3^gl^G	53.7	3.32	5.64	9.05
G	63.0	3.04	4.56	7.11

**Table 3:** Thermal degradation parameters obtained for G3^gl^G foam from TGA and DTG analysis.

	Residue (%)	T_5_% [℃]	T_10_% [℃]	T_50_% [℃]	T_1_ [℃]	T_2_ [℃]	T_3_ [℃]
Control	4.6	248.36	264.72	348.79	278.83	330.07	382.41
Gamma	14.76	251.18	269.12	362.17	288.92	339.18	381.01
EB	12.64	248.52	265.27	355.06	280.50	325.74	382.32

T_5_%, T_10_%, and T_50_%—temperatures corresponding to 5%, 10%, and 50% mass loss, respectively; T_1_, T_2_, and T_3_ temperatures corresponding to successive DTG peak maxima; EB—electron beam.

**Table 4:** Encapsulation efficiency (EE) and drug loading content (LC) of DOX determined by fluorescence-based mass balance.

Sample	LC [%]	EE [%]
G DOX	10.3	29.7
G3^gl^G DOX	12.0	34.9

**Table 5:** The influence of G and G3^gl^G on reconstructed human epidermis model estimated with MTT assay after 18 h incubation. Negative control: PBS without Mg^2+^ and Ca^2+^. Positive control: 5% sodium dodecyl sulfate (SDS).

SAMPLE	Mean Viability [%]	SD [%]	Result
Negative control	100	8.79	Correct
Positive control	2.1	0.16	Correct
G	91.9	3.54	No irritating effect
G3^gl^G	90.4	8.70	No irritating effect

## Data Availability

The original contributions presented in the study are included in the article/Supplementary Material. Further inquiries can be directed to the corresponding author.

## Supplementary Materials

The following supporting information can be downloaded at https://www.mdpi.com/article/10.3390/ma19214486/s1, Figure S1: Survey XPS spectrum of reference PU foam (G); Figure S2: Survey XPS spectrum of dendrimer-modified PU-PAMAM G3^gl^ foam (G3^gl^G); Table S1: Surface chemical composition as well as C 1s and N 1s deconvolution parameters for reference (G) and dendrimer-modified (G3^gl^G) polyurethane foams determined by XPS before and after ethanol extraction; Figure S3: TGA/DTG curves for sample G3^gl^G before and after irradiation with a dose of 25 kGy using electron beam (EB) and gamma radiation (Co-60). Analyses were performed in a nitrogen atmosphere with a temperature range of 30–800 °C at a heating rate of 10 °C min^−1^.
