# Supplementary material for: Polyurethane Foams Functionalized with Hydroxylated PAMAM G3 Dendrimers for Local Drug Delivery, Including Anticancer Therapy: In Vitro and In Vivo Studies
Source: Materials (Basel). 2026 Oct 24;19(21):4486. doi: 10.3390/ma19214486 (PMC13764446; doi:10.3390/ma19214486)
Supplement: Supplementary file 1 [file materials-19-04486-s001.zip › materials-4624840-supplementary.pdf]

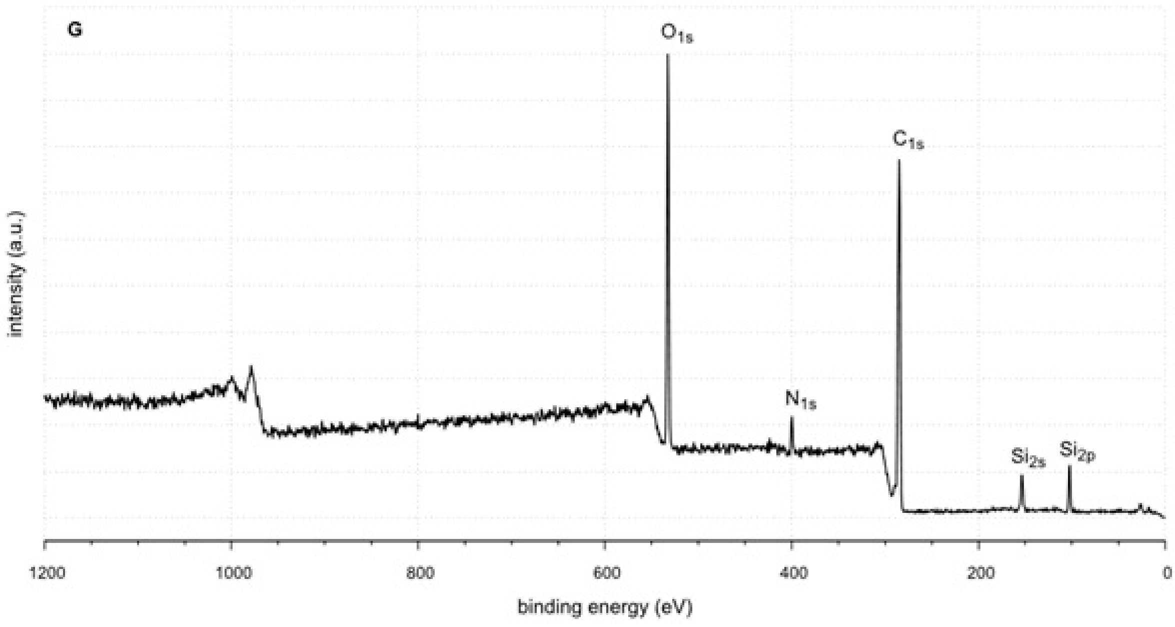

Figure S1. Survey XPS spectrum of reference PU foam (G).

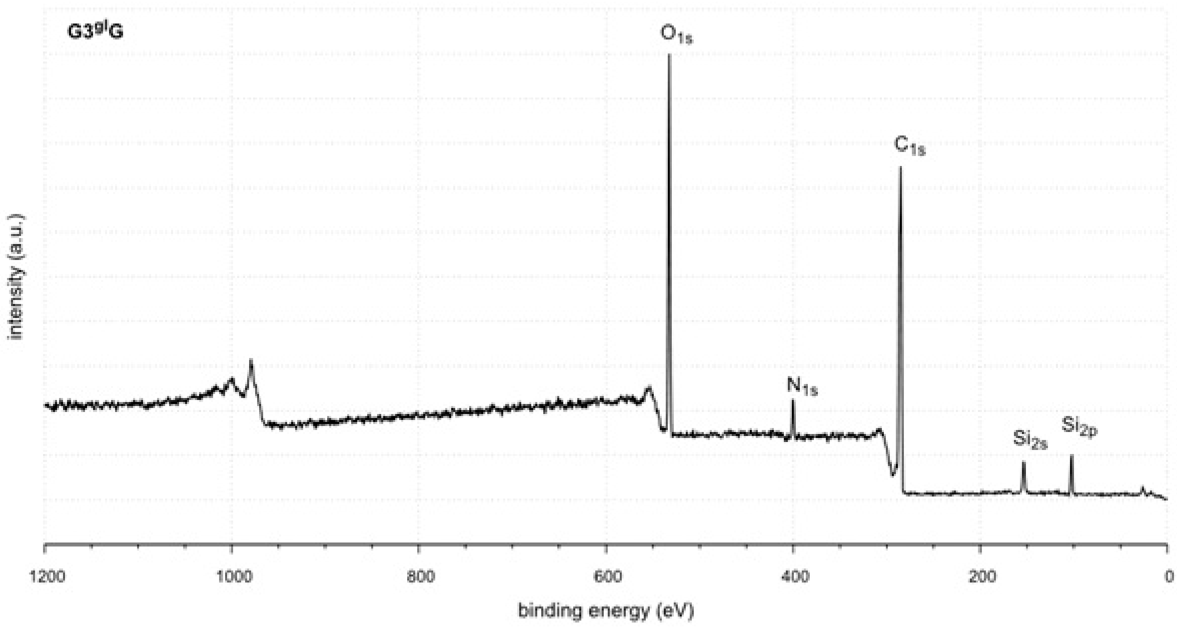

Figure S2. Survey XPS spectrum of dendrimer-modified PU-PAMAM G3^{gl} foam (G3glG).

Table S1. Surface chemical composition as well as C 1s and N 1s deconvolution parameters for reference (G) and dendrimer-modified (G3^{gl}G) polyurethane foams determined by XPS before and after ethanol extraction.

(A) Survey atomic composition

**Sample**	**C (at.%)**	**N (at.%)**	**O (at.%)**	**Si (at.%)**
G	68.1	3.9	19.7	8.3
G3^{gl}G	70.1	4.5	18.0	7.4
G3^{gl}G EtOH	73.7	3.8	18.7	3.9

(B) High-resolution C 1s deconvolution

**Sample**	**Component**	**BE (eV)**	**Contribution (%)**
G	C–C / C–H	285.00	44.8
	C–N	285.66	8.5
	C–O–C	286.73	42.6
	O–(C=O)–NH	289.61	4.1
G3^{gl}G	C–C / C–H	285.00	41.8
	C–N	285.66	11.9
	C–O–C	286.73	38.3
	C=O (dendrimer-associated)	287.81	3.4
	O–(C=O)–NH	289.59	4.7
G3^{gl}G EtOH	C–C / C–H	285.00	35.1
	C–N	285.66	13.5
	C–O–C	286.73	41.9
	C=O (dendrimer-associated)	287.82	4.6
	O–(C=O)–NH	289.58	4.9

(C) High-resolution N 1s deconvolution

Sample	Component	BE (eV)	Contribution (%)
G	C–NH	400.46	100
G3^{gl}G	C–NH	400.49	100

G3^{gl}G EtOH	C–NH	400.52	100

The atomic concentrations were determined from XPS survey spectra. The relative contributions of the C 1s and N 1s components were calculated from the corresponding high-resolution peak fitting. G3^{g}lG EtOH denotes the dendrimer-modified polyurethane foam after ethanol extraction.

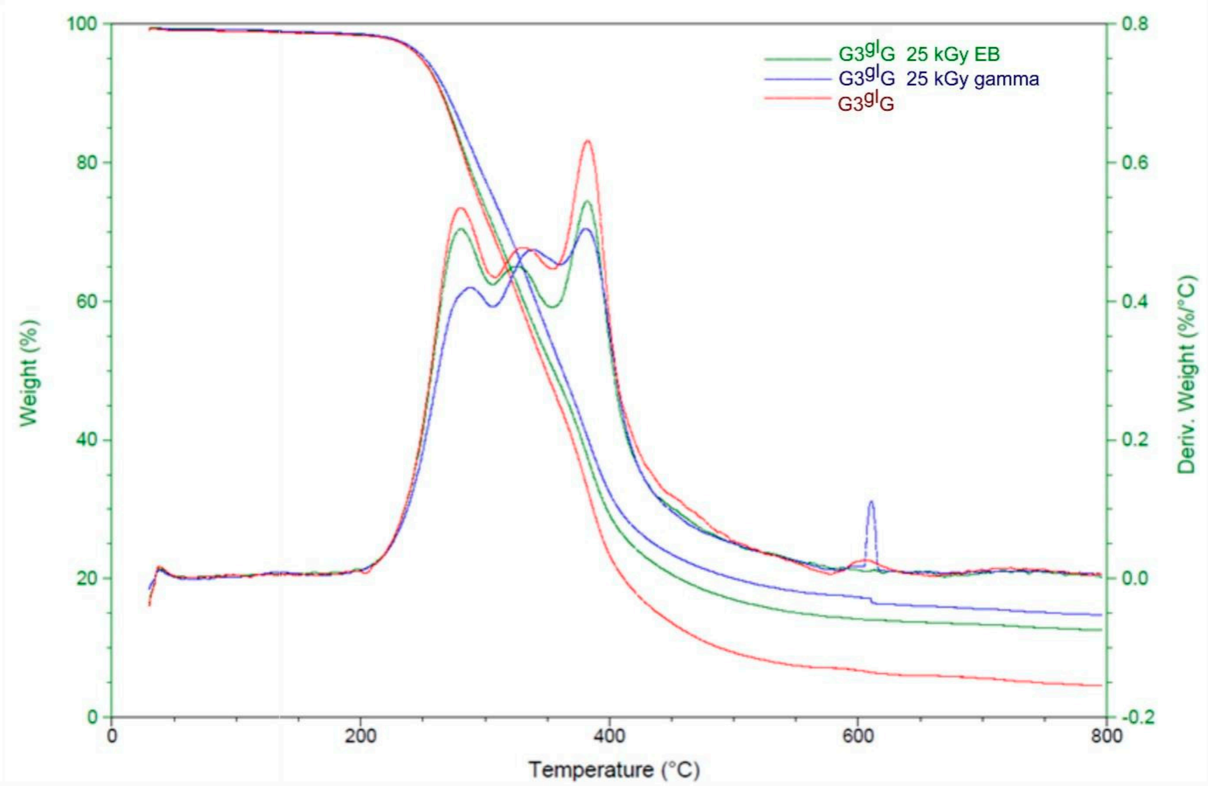

Figure S3. TGA/DTG curves for sample G3^{gl}G before and after irradiation with a dose of 25 kGy using electron beam (EB) and gamma radiation (Co-60). Analyses were performed in a nitrogen atmosphere with a temperature range of 30-800°C at a heating rate of 10 °C min^{-1}.
